# Maintenance of low inflammation level by the *ZFHX3* SNP rs2106261 minor allele contributes to reduced atrial fibrillation recurrence after pulmonary vein isolation

**DOI:** 10.1371/journal.pone.0203281

**Published:** 2018-09-04

**Authors:** Shunsuke Tomomori, Yukiko Nakano, Hidenori Ochi, Yuko Onohara, Akinori Sairaku, Takehito Tokuyama, Chikaaki Motoda, Hiroya Matsumura, Michitaka Amioka, Naoya Hironobe, Yousaku Ookubo, Shou Okamura, Hiroshi Kawazoe, Kazuaki Chayama, Yasuki Kihara

**Affiliations:** 1 Department of Cardiovascular Medicine, Hiroshima University Graduate School of Biomedical and Health Sciences, Hiroshima, Japan; 2 Department of Gastroenterology and Metabolism, Division of Frontier Medical Science, Programs for Biomedical Research Graduate School of Biomedical Science, Hiroshima University, Hiroshima, Japan; 3 Department of Internal Medicine, Chuden Hospital, The Chugoku Electric Power Company, Hiroshima, Japan; Indiana University, UNITED STATES

## Abstract

**Introduction:**

The single nucleotide polymorphism (SNP) rs2106261 in the transcription factor gene *ZFHX3* (16q22), a major regulator of inflammation, has been reported linking to atrial fibrillation (AF) by genome-wide association studies. Inflammation is known to be a strong predictor of atrial fibrillation recurrence after ablation, so we examined the association of the *ZFHX3* SNP rs2106261 to inflammation marker expression and recurrence after AF ablation.

**Methods:**

We genotyped *ZFHX3* SNP rs2106261 and compared the minor (T) allele frequency between 362 paroxysmal AF (PAF) patients underwent pulmonary vein isolation (PVI) and 627 non-AF controls. We also analyzed associations between *ZFHX3* SNP rs2106261 genotype and recurrence rate after pulmonary vein isolation and the inflammation markers.

**Results:**

The minor (T) allele frequency of the *ZFHX3* SNP rs2106261 was significantly higher in AF patients than non-AF controls (odds ratio 1.52, *p* = 2.2×10^−5^). Multivariable analysis revealed that the minor allele (T) decreased AF recurrence rate after pulmonary vein isolation (hazard ratio 0.53, *p* = 0.04). Further, neutrophil/lymphocyte (N/L) ratio, C-reactive protein (CRP), and interleukin-6 (IL-6) expression levels were lower in PAF patients with the *ZFHX3* SNP rs2106261 minor allele (TT+TC) than in CC patients (N/L ratio: CC 2.22 ± 0.08, TT+TC 1.98 ± 0.06, *p* = 0.018; CRP: CC 0.103 ± 0.009 mg/dl, TT+TC 0.076 ±0.007 mg/dl, *p* = 0.016; IL-6: CC 60.3 ± 3.0 pg/ml, TT+TC 52.8 ± 2.3 pg/ml, *p* = 0.04).

**Conclusions:**

The *ZFHX3* SNP rs2106261 minor allele is associated with lower AF recurrence rate after pulmonary vein isolation. Low baseline inflammation conferred by this allele may reduce AF recurrence risk.

## Introduction

Catheter ablation is a well established treatment strategy for patients with symptomatic atrial fibrillation (AF) [1]. Hypertension, left atrial dilatation, and non-paroxysmal AF are known predictors of recurrence after AF ablation [2, 3]. Markers of inflammation such as neutrophil/lymphocyte (N/L) ratio and C-reactive protein (CRP) were also associated with recurrence after AF ablation [4, 5]. In addition, a single nucleotide polymorphism (SNP) on chromosome 16q22 in the transcription factor gene *ZFHX3* (rs2106261, C>T, transition C to T) has been associated with AF by genome-wide association studies (GWASs) [6, 7]. The *ZFHX3* protein is a regulatory factor for STAT3-mediated signal transduction through its interaction with the protein inhibitor of activated STAT3, and STAT3 is an important mediator of the inflammatory process [8]. The contribution of the *ZFHX3* SNP rs2106261 to AF recurrence after catheter ablation has been examined but the results are controversial. One study reported that *ZFHX3* SNPs were associated with AF recurrence after catheter ablation [9], but another reported that SNPs in *ZFHX3* did not predict clinical recurrence after catheter ablation [10]. Here, we investigated the contribution of *ZFHX3* SNP rs2106261 to inflammation marker expression and recurrence after AF ablation.

## Material and methods

### Participants

This is a retrospective single-center study on the association between AF recurrence after AF ablation and *ZFHX3* SNP rs2106261 genotype. We enrolled 362 paroxysmal AF (PAF) patients (270 males and 92 females, mean age 62 ± 11 years) who underwent radiofrequency catheter ablation (RFCA) at Hiroshima University Hospital from November 2009 to July 2015 and 627 non-AF controls (313 males and 314 females, mean age 53 ± 10 years) from Hiroshima University Hospital. The control patients were all Japanese ethnicity and volunteers treated for diseases unrelated to heart function at Hiroshima University. Those with structural heart diseases or AF were excluded by interview. The Institutional Ethics Committee of the Graduate School of Biomedical Science at Hiroshima University approved all procedures involving human genome use. Written informed consent was obtained from all participants. All methods were performed in accordance with the relevant guidelines and regulations.

We replicated the GWAS-reported association of *ZFHX3* SNP rs2106261 with AF risk and compared the allele frequencies of this SNP between PAF subjects and non-AF controls. Subsequently, we analyzed the relationships between recurrence rate after first-time pulmonary vein isolation (PVI) and *ZFHX3* SNP rs2106261 genotype. We excluded 46 patients because they had received prior RFCA or had structural heart diseases, and ultimately 316 PAF patients were included for the analysis of recurrence rate. We also examined the relationships between *ZFHX3* SNP rs2106261 genotype and the inflammation markers N/L ratio, CRP, and interleukin-6(IL-6).

### Genotyping

Peripheral blood was obtained from all participants and genomic DNA extracted from leukocytes using the QIAamp DNA Blood Mini Kit (QIAGEN, Hilden, Germany) according to the standard protocol. We genotyped the *ZFHX3* SNP rs2106261 in all participants using TaqMan assays as previously described [11, 12]. For typing rs2106261, we used forward primer CGCGCCGAGGCCAACCATCCATTAAAATATCCAA and reverse primer ATGACGTGGCAGACTCAACCATCCATTAAAATATCCAAG. We also used Invader oligo GGATCTTGCATGGCCTCGTAGTGAGGTGAT, signal prove-G CGCGCCGAGGGACAATTCTCTGGACGAG, and signal prove-T ATGACGTGGCAGACAACAATTCTCTGGACGAGC.

### Echocardiography

Transthoracic echocardiography and transesophageal echocardiography were performed at our institution using a commercially available system (Vivid E9, GE Healthcare, Milwaukee, WI, USA or iE33, Philips Medical Systems, Andover, MA, USA) before RFCA. Experienced echocardiographers blinded to the genotyping results conducted all echocardiographic examinations and analyzed echocardiographic parameters [13].

### Electrophysiological study and RFCA

Anti-arrhythmia drugs (AADs) other than amiodarone were discontinued at least five half-lives before RFCA. PVI and bidirectional cavotricuspid isthmus (CTI) ablation were performed on all PAF subjects as previously reported [14]. Continuous PVI was performed using an open-irrigation 3.5-mm tip deflectable catheter (THERMOCOOL; Biosense Webster) under guidance of a 3D electro-anatomical mapping system (CARTO3, Biosense Webster) with computed tomography integration (CARTOMERGE, Biosense Webster) to achieve electrical isolation of the left- and right-side pulmonary veins. We confirmed PVI entrance and exit block, and then rechecked it under isoproterenol plus adenosine triphosphate infusion. We also performed a bidirectional CTI block with an endpoint of bidirectional conduction block in all patients following PVI.

After RFCA, an electrophysiological study was performed during stable sinus rhythm using three 5-French-gauge quadripolar electrode catheters, each with a 5-mm inter-electrode distance positioned at the high right atrium (HRA), His bundle, and right ventricle. The atrial signal to the His bundle (AH) and signal from the His bundle to the first ventricular activation interval (HV) were measured on the baseline intracardiac electrocardiography (ECG). The sinus node recovery time (SNRT) was measured as the recovery interval after 30-s stimulation from the HRA. The corrected SNRT (CSRT) was defined as the recovery interval in excess of the sinus cycle (i.e., CSRT = maxSNRT − sinus cycle length).

### Post-RFCA management and follow-up

We examined all AF patients in the outpatient clinic at 1, 3, 6, and 12 months after AF ablation. We checked whether they felt palpitation and chest discomfort, and performed ECG on every visit. If the patients felt palpitation, we lent them a portable electrocardiography monitor (ECG) for 1 month and checked for the presence of AF. The 24-h Holter recording combination with a portable ECG was performed at 3 and 6 months after AF ablation and every 6 months thereafter. We performed the same level of monitoring in all patients. Recurrence of AF was defined as a palpitation episodes lasting > 30 s or AF, atrial flutter, or atrial tachycardia episodes lasting > 30 s but excluded AF episodes within 3 months after RFCA (early recurrence) [15].

### Measurement of laboratory parameters

White blood cell (WBC) count and high-sensitivity CRP (hs-CRP) were measured one day before RFCA. The total WBC number and differential counts were measured using the XE-5000 system (Sysmex). The N/L ratio was computed as the absolute neutrophil count divided by the absolute lymphocyte count. Plasma levels of hs-CRP were determined by immunoturbidimetry (JCA-BM6070 BioMajesty, JEOL). Blood samples were also collected one day before RFCA to measure plasma IL-6 levels. The samples were centrifuged for 20 min at 4°C and the plasma was stored at −70°C until analysis. Plasma IL-6 levels (pg/ml) were determined by the immunoenzymatic method using a IL-6 Human ELISA Kit (Thermo Scientific, Germany).

### Statistical analysis

Normally distributed continuous variables are presented as mean ± standard deviation (SD). Group differences in continuous data were analyzed using the nonparametric Mann–Whitney *U* test. To test genetic associations for cases and controls, we used the χ^2^ test and Cochran–Armitage trend test. Deviation from Hardy–Weinberg equilibrium was tested among the cases and controls using an ordinary χ^2^ test. Differences in continuous data among genotypes were analyzed by linear regression. Odds ratios (ORs) and 95% confidence intervals are stated as appropriate. Multivariable analysis was performed using a Cox proportional hazards model. The chosen clinical covariates were based on expected relationships to AF recurrence after RFCA. A *p*-value of < 0.05 was considered significant for all tests significant.

## Results

### *ZFHX3* SNP rs2106261 genotypes in PAF patients and non-AF controls

The *ZFHX3* SNP rs2106261 was significantly associated with AF. Detailed results of the *ZFHX3* SNP rs2106261 genotypes in AF patients and non-AF controls are shown in S1 Table. The *ZFHX3* SNP rs2106261 MAF (T) was significantly greater in AF patients than non-AF controls (MAF 39% vs. 30%, OR 1.52, *p* = 2.2 × 10^−5^). Moreover, *ZFHX3* SNP rs2106261 presence was significantly associated with AF using dominant models.

### Relationship between *ZFHX3* SNP rs2106261 and PVI outcomes

**Fig 1:**
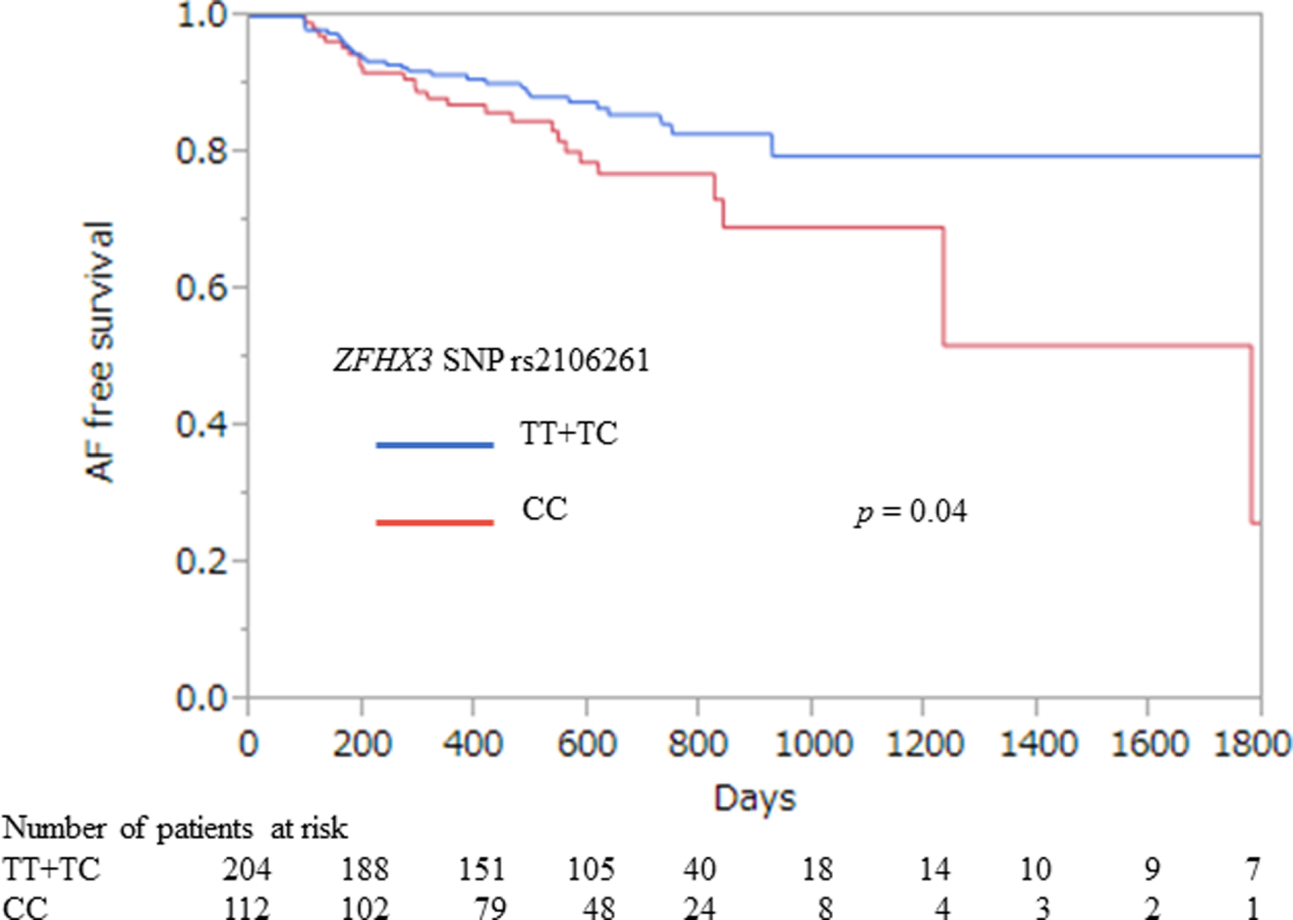
Kaplan–Meier analysis of atrial fibrillation (AF) time to recurrence according to *ZFHX3* SNP rs2106261 genotype for the dominant model. *ZFHX3* SNP rs2106261 minor allele genotype (TT or TC) was associated with lower atrial fibrillation recurrence rate after radiofrequency catheter ablation (*p* = 0.04).

There were no significant differences in clinical characteristics, echocardiographic parameters, and electrophysiological parameters among the three *ZFHX3* SNP rs2106261 genotypes (Table 1). Fig 1 shows Kaplan–Meier recurrence-free survival curves after RFCA. The AF recurrence rate after RFCA was lower in PAF patients with the *ZFHX3* SNP rs2106261 minor allele (TT or TC) than in CC patients (*p* = 0.04).

**Table 1:** Characteristics of PAF patients and *ZFHX3* SNP rs2106261.

	*ZFHX3* SNP rs2106261			
Genotype	CC	CT	TT	p values
No. of patients	131	181	50	
Clinical characteristics				
Age (years)	62.8 ± 10.9	62.2 ± 11.4	61.9 ± 11.2	0.825
Men (%)	93 (71.0)	141 (77.9)	36 (72.0)	0.346
Body mass index (kg/m^2^)	24.0 ± 3.7	23.7 ± 3.2	23.5 ± 2.9	0.578
Hypertension (%)	82 (63.1)	100 (55.6)	27 (54.0)	0.342
Diabetes (%)	23 (17.7)	28 (15.4)	4 (8.0)	0.226
Ischemic stroke (%)	4 (3.1)	18 (9.9)	4 (8.0)	0.086
Heart failure (%)	2 (1.5)	2 (1.1)	3 (6.0)	0.156
Ischemic heart disease (%)	7 (5.3)	5 (2.8)	1 (2.0)	0.389
Electrophysiological study				
AH (ms)	92.5 ± 26.2	98.2 ± 23.1	95.4 ± 19.2	0.204
HV (ms)	41.9 ± 8.7	40.3 ± 9.1	39.7 ± 10.7	0.106
maxSNRT (ms)	1292 ± 319	1312 ± 312	1286 ± 277	0.901
CSRT (ms)	444 ± 240	463 ± 220	465 ± 208	0.495
Echocardiography				
LAD (mm)	37.9 ± 6.1	38.1 ± 5.9	37.6 ± 5.8	0.918
EF (%)	61.4 ± 5.7	61.8 ± 5.6	62.6 ± 5.2	0.200
LVDd (mm)	47.9 ± 5.2	48.4 ± 4.5	48.1 ± 3.7	0.624
IVST (mm)	8.8 ± 1.7	8.9 ± 1.9	8.7 ± 1.3	0.973
LAV (ml)	64.6 ± 19.7	65.6 ± 18.3	64.7 ± 18.4	0.953
LAVI (ml/cm^2^)	37.6 ± 11.4	38.5 ± 10.7	37.7 ± 11.4	0.789
LAA area (mm^2^)	447 ± 147	459 ± 149	440 ± 145	0.976

CSRT; corrected sinus node recovery time, EF; ejection fraction,

IVST; interventricular septum thickness, LAA area; left atrial appendage area,

LAD; left atrial diameter, LAV; left atrial volume, LAVI; left atrial volume index,

LVDd; left ventricular diastolic diameter, PAF; paroxysmal atrial fibrillation,

SNP; single nucleotide polymorphism, SNRT; sinus node recovery time

Univariate analysis revealed that presence of the *ZFHX3* SNP rs2106261minor allele (TT or TC genotype) significantly decreased AF recurrence after RFCA [Table 2: hazard ratio (HR) = 0.57, *p* = 0.04]. Longer duration of AF and longer CSRT also tended to increase AF recurrence risk but these trends did not reach statistical significance. Multivariable analysis revealed that presence of the *ZFHX3* SNP rs2106261 minor allele (TT or TC) was independently associated with lower AF recurrence rate after RFCA (Table 2: HR = 0.53, *p* = 0.04).

**Table 2:** Clinical and genetic predictors of AF recurrence after PVI.

Variables	Univariate		Multivariate	
	HR (95%CI)	p values	HR (95%CI)	p values
Age (years)	0.99 (0.97–1.02)	0.66		
Sex (Men %)	0.73 (0.42–1.36)	0.31		
BMI (kg/m^2^)	1.01 (0.93–1.10)	0.73		
Duration of AF	1.00 (0.99–1.00)	0.09	1.00 (0.99–1.00)	0.31
Hypertension	1.15 (0.67–2.05)	0.61		
Diabetes	0.82 (0.34–1.70)	0.61		
CSRT (ms)	1.00 (0.99–1.00)	0.10	1.00 (0.99–1.00)	0.09
LAD (mm)	1.01 (0.96–1.06)	0.67		
EF (%)	1.00 (0.96–1.06)	0.83		
LAVI (ml/cm2)	1.01 (0.99–1.04)	0.33		
*ZFHX3* SNP rs2106261 dominant model	0.57 (0.33–0.99)	0.04	0.53 (0.29–0.98)	0.04

AF; atrial fibrillation, BMI; body mass index, CSRT; corrected sinus node recovery time,

EF; ejection fraction, HR; hazard ratio, LAD; left atrial diameter,

LAVI; left atrial volume index, PVI; pulmonary vein isolation

### Relationship between *ZFHX3* SNP rs2106261 and inflammation markers

**Fig 2:**
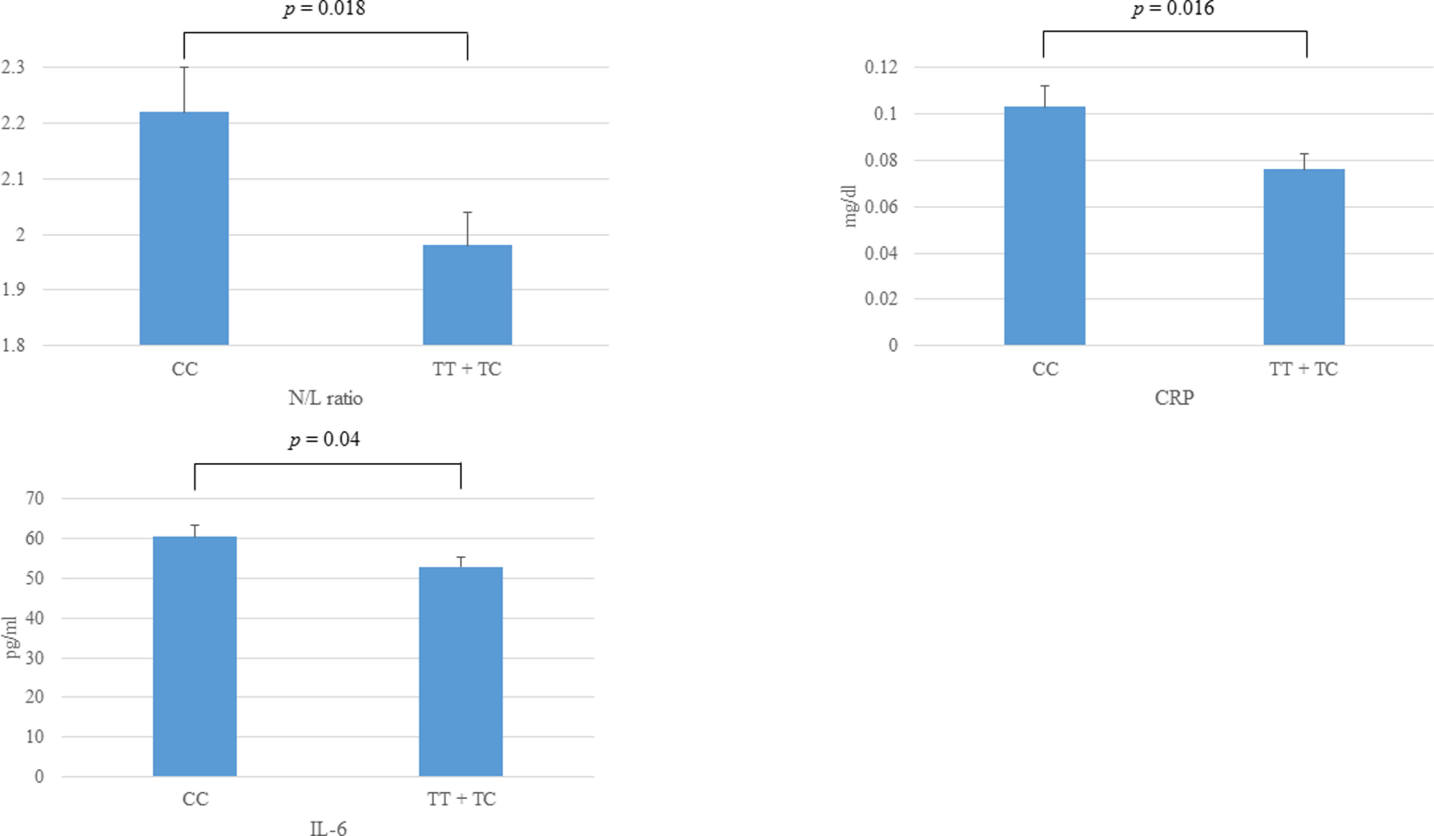
Relationship between ZFHX3 SNP rs2106261 genotype and the inflammation markers neutrophil/lymphocyte (N/L) ratio, C-reactive protein (CRP) and interleukin-6 (IL-6).

Fig 2 shows the relationships between *ZFHX3* SNP rs2106261 genotypes and the inflammation markers N/L ratio, CRP level, and plasma IL-6 level. All three were lower in patients with the *ZFHX3* SNP rs2106261 minor allele than in those without (N/L ratio: CC 2.22 ± 0.08, TT + TC 1.98 ± 0.06, *p* = 0.018; CRP: CC 0.103 ± 0.009 mg/dl, TT + TC 0.076 ± 0.007 mg/dl, *p* = 0.016; IL-6: CC 60.3 ± 3.0 pg/ml, TT + TC 52.8 ± 2.3 pg/ml, *p* = 0.04).

The N/L ratio, CRP level and plasma IL-6 level were lower in the TT + TC group than the CC group (N/L ratio: CC 2.22 ± 0.08, TT + TC 1.98 ± 0.06, *p* = 0.018; CRP: CC 0.103 ± 0.009 mg/dl, TT + TC 0.076 ± 0.007 mg/dl, *p* = 0.016; IL-6: CC 60.3 ± 3.0 pg/ml, TT + TC 52.8 ± 2.3 pg/ml, *p* = 0.04).

## Discussion

In our study, we confirmed the previously reported association between AF and *ZFHX3* SNP rs2106261 by GWAS [7] in Japanese PAF patients at our institute. More importantly, we revealed that PAF patients with the *ZFHX3* SNP rs2106261 minor allele (T) were more likely to maintain sinus rhythm after RFCA.

We also found that inflammation markers (N/L ratio, CRP, and IL-6) were lower in PAF patients with the *ZFHX3* SNP rs2106261 minor allele (TT or TC genotype) than in those without (CC genotype).

Husser et al. reported that the *ZFHX3* gene SNP rs12373097 was associated with AF recurrence in both PAF and persistent AF patients [9], while Park et al. reported that the *ZFHX3* gene SNP rs2106261 was an independent predictor of good responders to RFCA among long-standing persistent AF patients [16]. However, both Shoemaker et al. and Choi et al. reported that *ZFHX3* polymorphisms did not predict clinical recurrence of AF after catheter ablation among PAF and persistent AF patients [10, 17]. Thus, the contributions of *ZFHX3* SNPs to AF recurrence after RFCA remain controversial. Although different SNPs (rs12373097 vs. rs2106261) may account for the discrepancy, cohort and treatment heterogeneity may also contribute. First, these previous studies analyzed recurrence rate in a combined cohort of persistent AF and PAF patients. Second, strategies for RFCA are multifarious, especially in persistent AF patients. In the present study, we enrolled only PAF patients schedule for first AF RFCA and the treatment strategy was PVI exclusively. In this restricted cohort, we found that the *ZFHX3* SNP rs2106261 minor allele (T) was independently associated with low AF recurrence rate.

A systematic review of predictors for atrial fibrillation recurrence after ablation [18] reported that EF and LAD parameters are not independent predictors of AF recurrence among patients with approximately normal EF and LAD. In the present study as well, EF and LAD were not independent predictors of AF recurrence because all AF types were paroxysmal, average EF was normal, and average LAD was not markedly elevated (EF = 61.7% ± 5.6%, LAD = 37.9 ± 5.9 mm).

In this study, we revealed that the *ZFHX3* SNP rs2106261 minor allele (T) is associated with high AF occurrence but low recurrence after AF ablation. The *ZFHX3* SNP rs2106261 was linked to AF onset by a GWAS, but the precise mechanism of association with AF pathogenesis has not been elucidated. Similarly, the mechanisms underlying this discrepancy between onset risk and recurrence risk remain to be revealed. The minor allele of *ZFHX3* SNP rs2106261 was also associated with high BMI [19], but in our study BMI was similar among *ZFHX3* SNP rs2106261 genotypes. *ZFHX3* knockdown in atrial myocytes was reported to dysregulate calcium homeostasis and increase atrial arrhythmogenesis, ultimately contributing to AF occurrence [20]. Huang et al. reported that interaction between *ZFHX3* SNP and *PITX2* SNP. They also reported that *ZFHX3* positively regulated *PITX2c* expression [21]. The *PITX2* regulates process that pulmonary mesenchyme differentiates in myocardium, initiates a phase of rapid proliferation and expands to form the myocardial sheet around the pulmonary vein branches. Overexpression of *PITX2* may promote the proliferation of myocardial sleeve around the pulmonary vein branches and increase automaticity from there [22]. *ZFHX3* SNP rs2106261 may modulate *PITX2c* expression levels and associate with high occurrence of AF. We performed PV isolation and paroxysmal AF patients with *ZFHX3* SNP minor allele have lower AF recurrence rate than those without. Given that *ZFHX3* SNP acts through *PITX2c* expression levels, AF trigger in paroxysmal AF patients with *ZFHX3* SNP minor allele was related to PV and they easily cured by PVI.

We also reported in this paper that the *ZFHX3* SNP rs2106261 minor allele is associated with low baseline inflammation levels. There are many reports that inflammatory factors are involved in AF occurrence and recurrence [23, 24]. However, Wu et al. reported that CRP level was higher in patents with persistent AF but similar in patients with paroxysmal AF compared to controls [25]. Lin et al. reported that higher pre-ablation hs-CRP was associated with an abnormal left atrial substrate [26]. In paroxysmal AF patients, triggers have the greatest impact on AF occurrence. However, once pulmonary vein isolation was performed, low inflammation levels conferred by the *ZFHX3* SNP rs2106261 minor allele may reduce the risk of recurrence. If the paroxysmal AF patients possess the ZFHX3 SNP minor allele, we can preprocedurely select only PVI as AF ablation strategy.

## Conclusion

The *ZFHX3* SNP rs2106261 was associated with PAF among Japanese patients in our replication study. The *ZFHX3* SNP rs2106261 minor allele (T) was associated with lower AF recurrence rate after PVI, and low baseline inflammation may contribute to this reduced recurrence risk. The *ZFHX3* SNP rs2106261 may be an important genetic marker for prediction of AF non-recurrence after AF RFCA and a useful guide for selecting therapeutic interventions.

## Supporting information

S1 Table *ZFHX3* SNP polymorphism (rs2106261) in patients with AF and non-AF controls. (DOCX)
