# Supplementary material for: Maintenance of low inflammation level by the ZFHX3 SNP rs2106261 minor allele contributes to reduced atrial fibrillation recurrence after pulmonary vein isolation
Source: PLoS One. 2018 Sep 4;13(9):e0203281. doi: 10.1371/journal.pone.0203281 (PMC6122824; doi:10.1371/journal.pone.0203281)
Supplement: S1 Table — (DOCX) [file pone.0203281.s001.docx]

|  | Genotype distribution | | |  | MAF |  | HW test |  | Allelic Model (CvsT) | |  | Dominant Model | |  | Recessive Model | |
| --- | --- | --- | --- | --- | --- | --- | --- | --- | --- | --- | --- | --- | --- | --- | --- | --- |
|  |  |  |  |  |  |  |  |  |  |  |  | (TT+TCvsCC) | |  | (TTvsTC+CC) | |
|  | CC | TC | TT |  |  |  | p |  | p | OR(95%CI) |  | p | OR(95%CI) |  | p | OR(95%CI) |
| AF | 131 (36.0%) | 181 (50.0%) | 50 (14.0%) |  | 0.39 |  | 0.32 |  | 2.2×10^-5^ | 1.5 (1.2-1.8) |  | 1.2×10^-5^ | 1.8 (1.4-2.4) |  | 4.0×10^-2^ | 1.5 (1.0-2.3) |
| Control | 317 (48.7%) | 250 (42.0%) | 60 (9.3%) |  | 0.30 |  | 0.30 |  |  |  |  |  |  |  |  |  |
|  |  |  |  |  |  |  |  |  |  |  |  |  |  |  |  |  |
| The C allele was considered as the reference allele in the allelic model. | | | | | | | | | |  |  |  |  |  |  |  |
| The results were tested by the chi-square test and the Cochran–Armitage trend test. | | | | | | | | | | |  |  |  |  |  |  |
| CI; confidence interval, HW; Hardy-Weinberg, MAF; minor allele frequency, OR; odds ratio | | | | | | | | | | |  |  |  |  |  |  |
